# KinESim: Pre-equilibrium kinetic simulation of electrochemical reactions

**DOI:** 10.21105/joss.01532

**Published:** 2019-08-23

**Authors:** Christopher W. John, Denis A. Proshlyakov

**Affiliations:** 1 Department of Chemistry, Michigan State University

## Summary

### Rationale:

Electrochemistry studies reduction-oxidation reactions, or reactions that involve transfer of an electron, with a particular emphasis on the reactions occurring at the interface between the electrode and the solution. Multiple electrochemical techniques can provide valuable insight into the thermodynamic properties of analytes and their chemical mechanisms. Extensive efforts have been invested into developing a quantitative description of the dynamics of electron transfer and ensuing mass transport where the analyte itself can undergo direct reduction/oxidation on the surface of the electrode. However, more complex cases where a redox-active analyte requires one or more redox-active mediators to transfer an electron to/from the electrode received less attention. Additional complexity may arise from the kinetic limitations of such multi-step reactions and the inherent nature of electrochemistry: it relies on the detection of electric currents, which report on the rate of a chemical process, while often focusing on the thermodynamic properties of the analyte, which represents equilibrium conditions. This conundrum requires a balance between detectability (fast processes) and accuracy (slow processes). Therefore, in most cases electrochemical studies are conducted under pre-equilibrium conditions.

KinESim is a procedure package for Igor Pro that was primarily designed to predict pre-equilibrium concentrations and their changes in a multi-component, redox-active chemical mixture. In general, this includes homogenous electrochemical reactions in the solution and heterogenous processes on the solution/electrode boundary. Heterogenous electrochemical reactions are controlled by an applied electric potential of an arbitrary waveform (continuous or pulsed). This allows one to use KinESim to predict pre-equilibrium changes in a variety of standard techniques, such as pulse, staircase, or cyclic voltammetry, as well as custom potential profiles. KinESim supports branching, sequential, and isomerization reactions in various stochiometric ratios over both phases. Unlike experimental electrochemical methods that report net electric current/charge, KinESim provides an insight into changes occurring to the individual reagents and their redox sub-population. This is particularly significant for spectro-electrochemical and indirect (mediated) electrochemical studies where redox changes in the analytes of interests may be difficult to detect from the electric current alone. Therefore, it can be used to predict experimental data and to test proposed reaction mechanisms in most chemical processes controlled by a combination of kinetics and thermodynamics. These abilities have recently been demonstrated in a study of how electrochemical mediators affect the observed electrochemical properties of an analyte (John & Proshlyakov, 2019; John, Swain, Hausinger, & Proshlyakov, 2019).

### Concept:

KinESim is suitable for modeling reactions in a thin solution layer over an electrode. It distinguishes two phases, electrode and solution, with mass transport between them. The current version makes an assumption that diffusion within the solution, in the direction perpendicular to the electrode, is fast relative to the process being modeled. In other words, the concentration of any analyte throughout the solution is uniform. KinESim implements a deterministic kinetics model for a continuous-time Markov process (Anderson & Kurtz, 2011; C. Zhang et al., 2019). Reactions in the solution and on the electrode, as well as adsorption/desorption processes are described by a system of ordinary differential equation (ODEs) derived from differential rate laws. Numerical integration of such system of ODEs using initial conditions, reaction order, and rate or equilibrium constants provided by the user yields time-dependent concentrations of all components. In addition, potential applied to the electrode controls electrochemical kinetics per the Butler-Volmer equation.

ODEs are integrated using the 4th-order Runge-Kutta method. This integration method includes a built-in adaptive timing method to minimize both the amount of error in each calculation and the amount of time needed to complete the simulation. The adaptive timing algorithm is particularly beneficial when KinESim is used with nonmonotonic processes. Since the simulation makes no assumption about the applied potential waveform, it gives the user full flexibility in the experimental design at the expense of computational uncertainty in the future progression of the reaction (which distinguishes KinESim from traditional simulations of chemical kinetics). Balance between computational time and accuracy is achieved by establishing boundary conditions for relative concentration changes. This allows for dynamic changes in the simulation time granularity if the chemical system is rapidly shifted off-equilibrium by large changes in the applied potential.

## Supplementary Material

Software archive
