# Supplementary figures and images for: KinESim: Pre-equilibrium kinetic simulation of electrochemical reactions
Source: J Open Source Softw. Author manuscript; Available in PMC 2020 Mar 2. (PMC7051063; doi:10.21105/joss.01532)

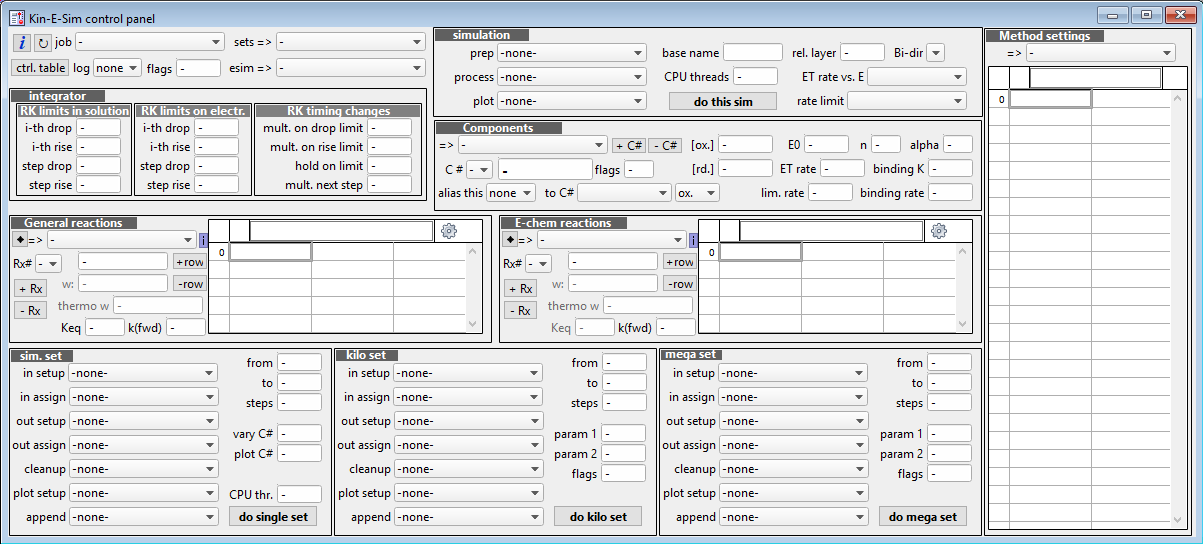

Supplement: Software archive [file NIHMS1559314-supplement-Software_archive.zip › KinESim-master/Docs/Figures/ControlPanel.png]

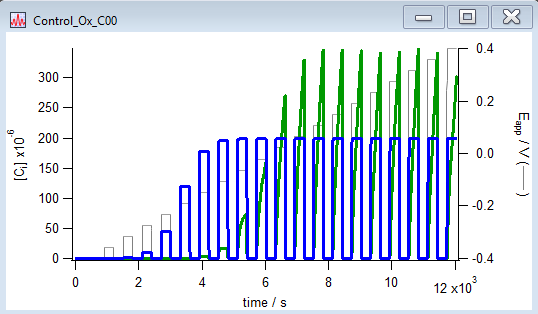

Supplement: Software archive [file NIHMS1559314-supplement-Software_archive.zip › KinESim-master/Docs/Figures/Control_Ox_C00.png]

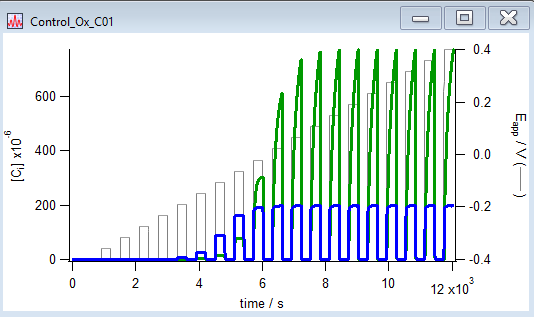

Supplement: Software archive [file NIHMS1559314-supplement-Software_archive.zip › KinESim-master/Docs/Figures/Control_Ox_C01.png]

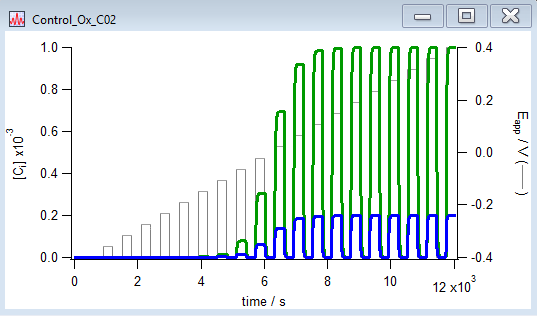

Supplement: Software archive [file NIHMS1559314-supplement-Software_archive.zip › KinESim-master/Docs/Figures/Control_Ox_C02.png]

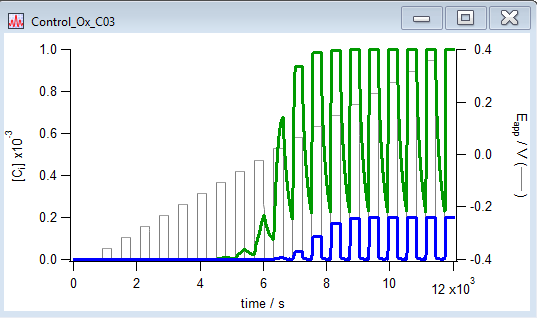

Supplement: Software archive [file NIHMS1559314-supplement-Software_archive.zip › KinESim-master/Docs/Figures/Control_Ox_C03.png]

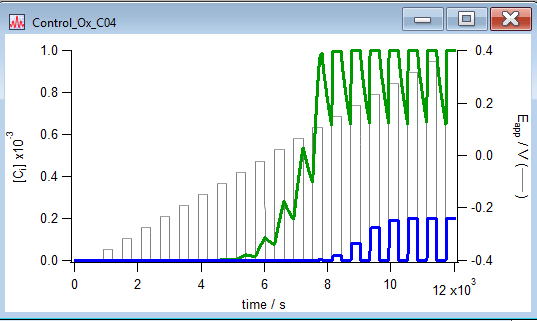

Supplement: Software archive [file NIHMS1559314-supplement-Software_archive.zip › KinESim-master/Docs/Figures/Control_Ox_C04.png]

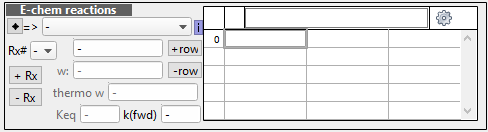

Supplement: Software archive [file NIHMS1559314-supplement-Software_archive.zip › KinESim-master/Docs/Figures/EchemRxns_sp.png]

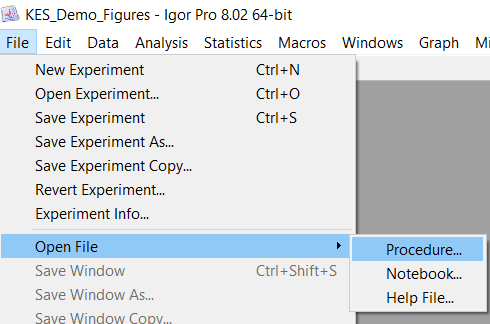

Supplement: Software archive [file NIHMS1559314-supplement-Software_archive.zip › KinESim-master/Docs/Figures/File_Open_Procedure.png]

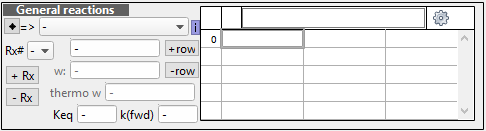

Supplement: Software archive [file NIHMS1559314-supplement-Software_archive.zip › KinESim-master/Docs/Figures/GRxns_sp.png]

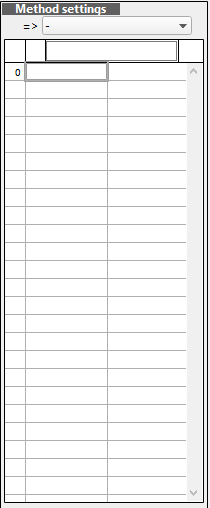

Supplement: Software archive [file NIHMS1559314-supplement-Software_archive.zip › KinESim-master/Docs/Figures/MethodSettings_sp.png]

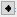

Supplement: Software archive [file NIHMS1559314-supplement-Software_archive.zip › KinESim-master/Docs/Figures/Rxns_create.png]

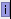

Supplement: Software archive [file NIHMS1559314-supplement-Software_archive.zip › KinESim-master/Docs/Figures/Rxns_sp_i.png]

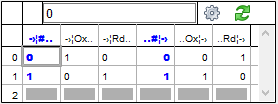

Supplement: Software archive [file NIHMS1559314-supplement-Software_archive.zip › KinESim-master/Docs/Figures/Rxns_table.png]

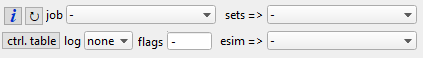

Supplement: Software archive [file NIHMS1559314-supplement-Software_archive.zip › KinESim-master/Docs/Figures/TLC.png]

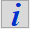

Supplement: Software archive [file NIHMS1559314-supplement-Software_archive.zip › KinESim-master/Docs/Figures/TLC_i.png]

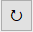

Supplement: Software archive [file NIHMS1559314-supplement-Software_archive.zip › KinESim-master/Docs/Figures/TLC_refresh.png]

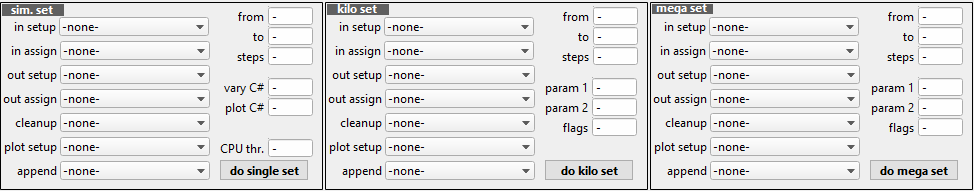

Supplement: Software archive [file NIHMS1559314-supplement-Software_archive.zip › KinESim-master/Docs/Figures/allsets_sp.png]

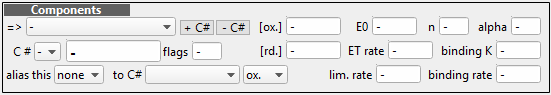

Supplement: Software archive [file NIHMS1559314-supplement-Software_archive.zip › KinESim-master/Docs/Figures/components_sp.png]

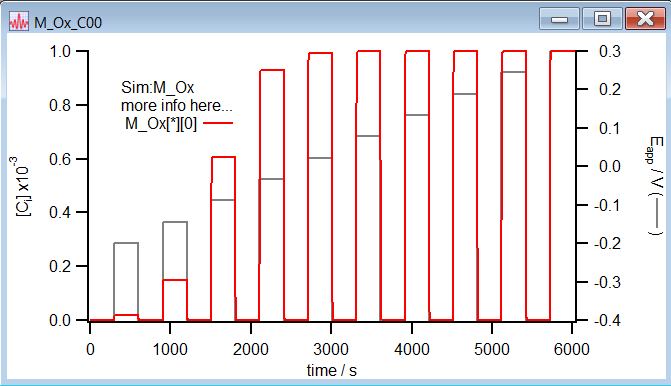

Supplement: Software archive [file NIHMS1559314-supplement-Software_archive.zip › KinESim-master/Docs/Figures/demoFig1.png]

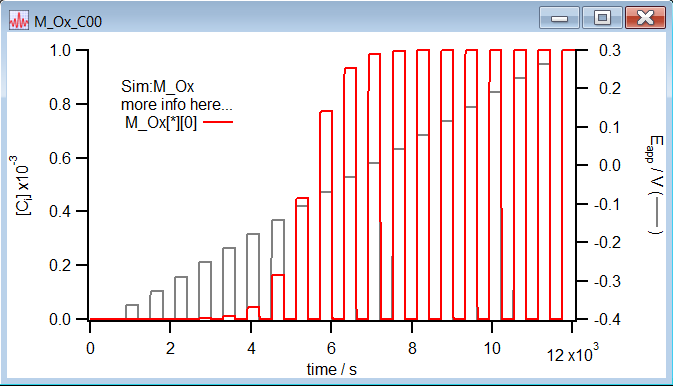

Supplement: Software archive [file NIHMS1559314-supplement-Software_archive.zip › KinESim-master/Docs/Figures/demoFig2.png]

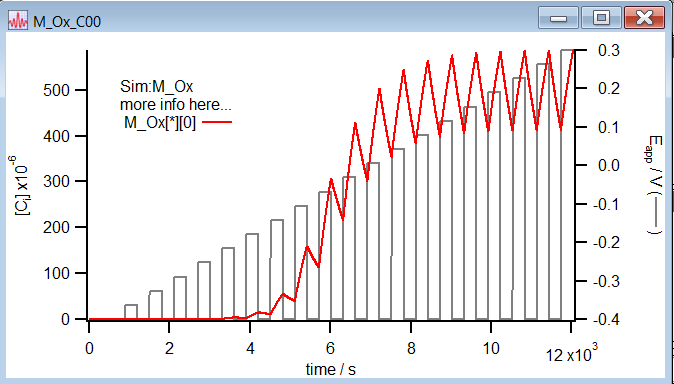

Supplement: Software archive [file NIHMS1559314-supplement-Software_archive.zip › KinESim-master/Docs/Figures/demoFig3.png]

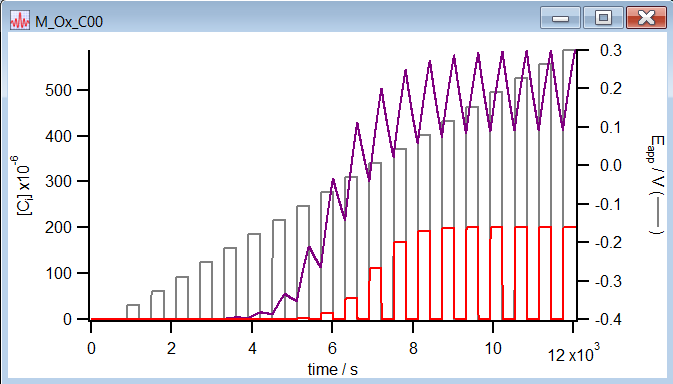

Supplement: Software archive [file NIHMS1559314-supplement-Software_archive.zip › KinESim-master/Docs/Figures/demoFig4.png]

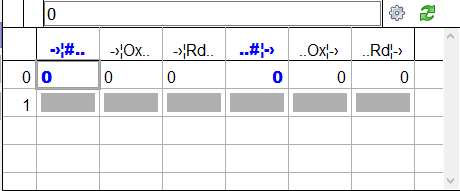

Supplement: Software archive [file NIHMS1559314-supplement-Software_archive.zip › KinESim-master/Docs/Figures/demoFig5.png]

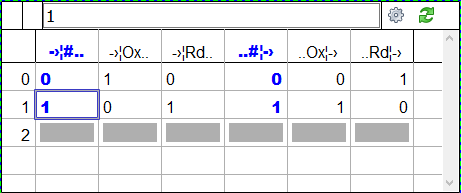

Supplement: Software archive [file NIHMS1559314-supplement-Software_archive.zip › KinESim-master/Docs/Figures/demoFig6.png]

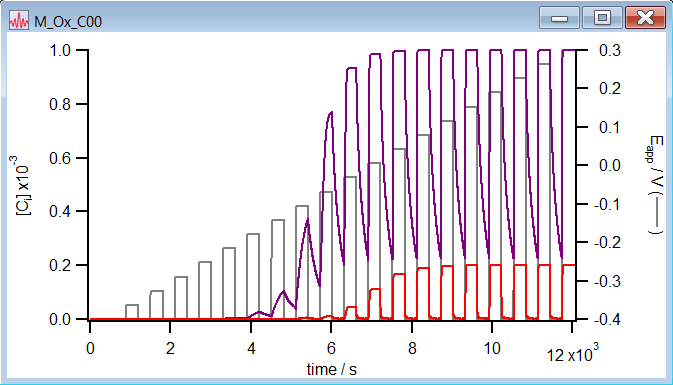

Supplement: Software archive [file NIHMS1559314-supplement-Software_archive.zip › KinESim-master/Docs/Figures/demoFig7.png]

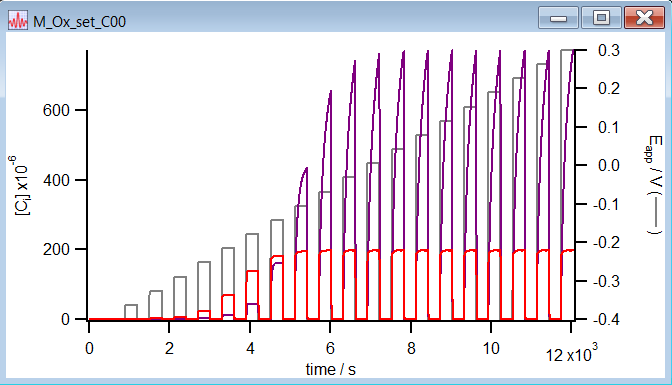

Supplement: Software archive [file NIHMS1559314-supplement-Software_archive.zip › KinESim-master/Docs/Figures/demoFig8a.png]

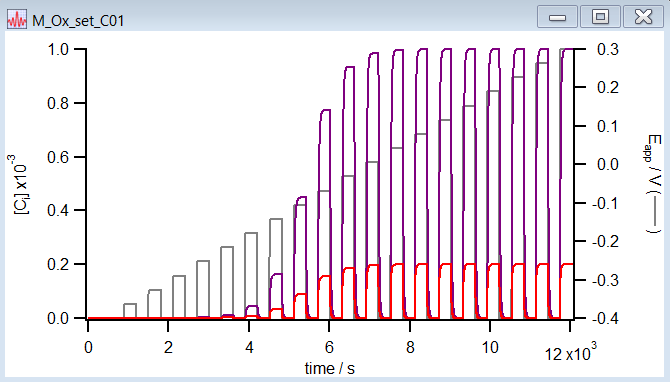

Supplement: Software archive [file NIHMS1559314-supplement-Software_archive.zip › KinESim-master/Docs/Figures/demoFig8b.png]

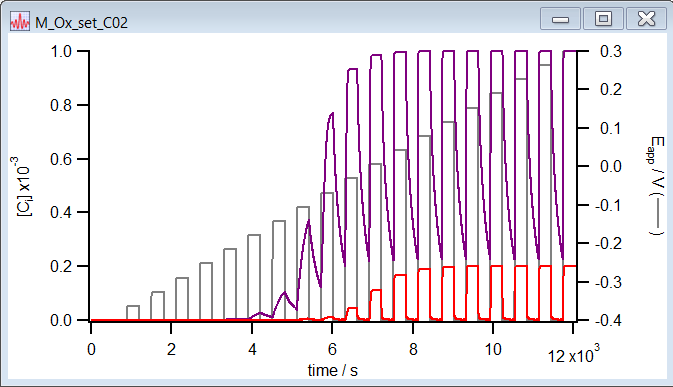

Supplement: Software archive [file NIHMS1559314-supplement-Software_archive.zip › KinESim-master/Docs/Figures/demoFig8c.png]

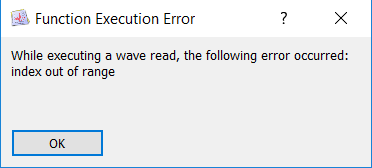

Supplement: Software archive [file NIHMS1559314-supplement-Software_archive.zip › KinESim-master/Docs/Figures/error.png]

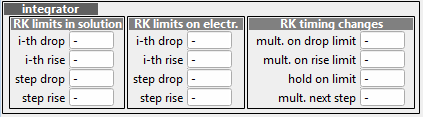

Supplement: Software archive [file NIHMS1559314-supplement-Software_archive.zip › KinESim-master/Docs/Figures/integrator_sp.png]

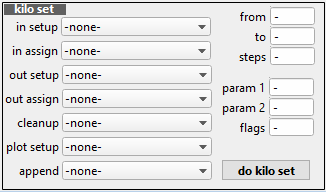

Supplement: Software archive [file NIHMS1559314-supplement-Software_archive.zip › KinESim-master/Docs/Figures/kiloset_sp.png]

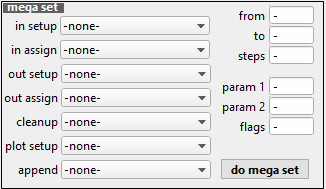

Supplement: Software archive [file NIHMS1559314-supplement-Software_archive.zip › KinESim-master/Docs/Figures/megaset_sp.png]

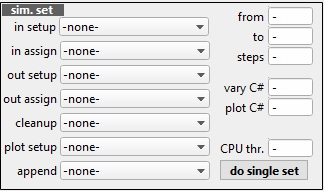

Supplement: Software archive [file NIHMS1559314-supplement-Software_archive.zip › KinESim-master/Docs/Figures/simset_sp.png]

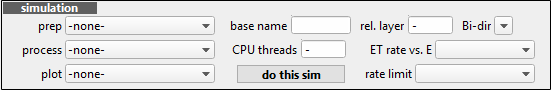

Supplement: Software archive [file NIHMS1559314-supplement-Software_archive.zip › KinESim-master/Docs/Figures/simulation_sp.png]
